# App-Based Evaluation of Older People’s Fall Risk Using the mHealth App Lindera Mobility Analysis: Exploratory Study

**DOI:** 10.2196/36872

**Published:** 2022-08-16

**Authors:** Nicole Strutz, Hanna Brodowski, Joern Kiselev, Anika Heimann-Steinert, Ursula Müller-Werdan

**Affiliations:** 1 Geriatrics Research Group Charité - Universitätsmedizin Berlin (corporate member of Freie Universität Berlin and Humboldt-Universität zu Berlin) Berlin Germany; 2 Department of Physiotherapy, Pain and Exercise Research Lübeck Institute of Health Sciences University of Lübeck Lübeck Germany; 3 Department of Anesthesiology and Operative Intensive Care Medicine (Charité Campus Virchow Clinic/Campus Charité Mitte) Charité - Universitätsmedizin Berlin (corporate member of Freie Universität Berlin and Humboldt-Universität zu Berlin) Berlin Germany

**Keywords:** mobility, fall risk, smartphone, app, analysis, older people, accuracy, mobility restriction

## Abstract

**Background:**

Falls and the risk of falling in older people pose a high risk for losing independence. As the risk of falling progresses over time, it is often not adequately diagnosed due to the long intervals between contacts with health care professionals. This leads to the risk of falling being not properly detected until the first fall. App-based software able to screen fall risks of older adults and to monitor the progress and presence of fall risk factors could detect a developing fall risk at an early stage prior to the first fall. As smartphones become more common in the elderly population, this approach is easily available and feasible.

**Objective:**

The aim of the study is to evaluate the app Lindera Mobility Analysis (LIN). The reference standards determined the risk of falling and validated functional assessments of mobility.

**Methods:**

The LIN app was utilized in home- and community-dwelling older adults aged 65 years or more. The Berg Balance Scale (BBS), the Tinetti Test (TIN), and the Timed Up & Go Test (TUG) were used as reference standards. In addition to descriptive statistics, data correlation and the comparison of the mean difference of analog measures (reference standards) and digital measures were tested. Spearman rank correlation analysis was performed and Bland-Altman (B-A) plots drawn.

**Results:**

Data of 42 participants could be obtained (n=25, 59.5%, women). There was a significant correlation between the LIN app and the BBS (*r*=–0.587, *P*<.001), TUG (*r*=0.474, *P*=.002), and TIN (*r*=–0.464, *P*=.002). B-A plots showed only few data points outside the predefined limits of agreement (LOA) when combining functional tests and results of LIN.

**Conclusions:**

The digital app LIN has the potential to detect the risk of falling in older people. Further steps in establishing the validity of the LIN app should include its clinical applicability.

**Trial Registration:**

German Clinical Trials Register DRKS00025352; https://tinyurl.com/65awrd6a

## Introduction

As part of the aging process, older adults are affected by an increasing risk of falling as well as accidental falls [1]. In Europe, this development leads to fall incidence rates for older adults aged 70 years or more between 7500 and nearly 20,000 falls per 100,000 inhabitants and a death rate of up to 153.2 per 100,000 inhabitants [2]. In a study by Choi et al [3], observed fall-related injury locations in older adults (≥60 years, n=1840) included lower and upper extremities (32.06% and 23.12%, respectively) but also 15.26% of falls resulting in head injuries, while 30.9% suffered 1 or more fractures. Additionally, falls and the risk of falling have a variety of effects on older adults’ attitudes and behavior. Falls and even the risk of falling can pose a high risk of losing independence [4]. The risk of falls in older people changes over time as health status [5] or medication [6], either prescribed by a doctor or self-medication, changes. Often, the risk of falls increases with age-related decline in body musculature [7] and overall decrease in functional performance [8]. The risk of falling develops over time, and it is often underdiagnosed [9]. Therefore, the risk of falling is often not properly detected until the first fall. One possible solution to this dilemma is a more frequent self-assessment that should start before the first fall. Technology-based assessments of fall risk can assist an older adult in assessing their own fall risk. In this area, analyzing gait patterns is a widely used strategy to track the progress of functional abilities and to assess the risk of falling. However, gait analysis systems, such as GAITRite or SensFloor, cannot be applied at home with minimal effort. In contrast, as mobile phones become more widespread in the elderly population [10], an app-based fall risk assessment would be easily available and feasible. Mobile applicable apps, such as FallSA (a fall risk–screening app) [11] and Lindera Mobility Analysis (LIN; Lindera GmbH, Berlin, Germany) [12], 2 commercially available apps, are location independent and applicable at home.

As scientific evidence on the validity of such apps is limited, the aim of this explorative study was to evaluate the app LIN in comparison to established and validated functional assessments of mobility as a reference standard.

## Methods

### Study Design and Ethical Considerations

In 2021, this explorative validation study was conducted in Germany by the Geriatrics Research Group of Charité – Universitätsmedizin Berlin. The study was approved by the Ethics Committee of Charité – Universitätsmedizin Berlin (#EA1/363/20; date of approval: April 4, 2021). A sample size calculation was not performed as the study was exploratory in nature.

### Recruitment

Participants were recruited from 3 sources: (1) the Geriatrics Research Group database, comprising older people who gave their consent to be contacted for participation in research projects; (2) older people who were staying in a geriatric hospital or day-care facility; and (3) a group of nursing home residents. Contact was made by mail, telephone, or a personal interview on-site. Inclusion criteria were age 65 years or older, being able to walk, and getting up from a chair and sitting down again. Participants were allowed to use walking aids, such as a wheeled walker or crutches. Exclusion criteria were defined as any fall events in the week before recruitment, more than 3 fall events during the past 6 months, and incapability of giving consent.

### Data Collection

Data collection was conducted in the laboratory of the Geriatrics Research Group as well as in a nursing home and 2 day-care facilities. In addition to sociodemographic data, such as age and gender, the care level, degree of disability, data of mobility, and fall risk of the participants were recorded. The official care level within the German health care system ranges from level 0 (no need for care) to level 5 (maximum need for care)—§61b (1) German Social Code (SGB) XII, where SGB refers to the German Social Code. The official level of disability is characterized by level 20 (low disability) to level 100 (maximum disability)—§2 SGB IX. In addition, 4 mobility tests were performed, 3 reference assessments and LIN. In all measurements, LIN was used first. For this, participants filled out the app’s questionnaire independently or, if preferred, together with the researcher. A video of the patient’s gait was recorded using LIN on a smartphone. In a second step, 3 reference assessments were used to test the participants’ fall risk and mobility restrictions. Between assessments, the participants could rest by answering the questionnaire on sociodemographic data. All data were collected within 1 session.

### Lindera Mobility Analysis

LIN version 10.3.0 was used to determine the fall risk by computing a fall risk score. Input parameters to compute the fall risk score included (1) video analysis of each participant’s gait through an artificial intelligence–based algorithm [13] and (2)a standardized questionnaire on further fall risk factors.

**Figure 1:**
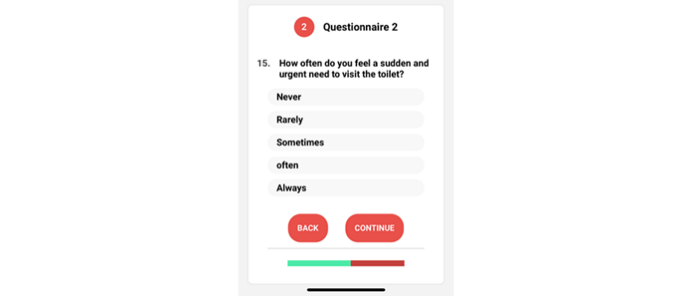
Questionnaire: example of a person-related risk factor.

The assessment was conducted with a mobile app using a smartphone with an integrated camera. The fall risk score is the weighted sum of 14 fall risk factors, as defined by the German National Expert Standard Fall Prevention [14], a guideline developed and published by German Network for Quality Development in Nursing (DNQP) [15]. The standardized questionnaire addresses both person-related risk factors, such as polypharmacy, diseases, or alcohol consumption (“How often do you consume alcoholic beverages during the week: not at all, 1x-2x/week, 3x-5x/week, or 6x-7x/week?”) and incontinence (“How often do you feel a sudden and urgent need to visit the toilet: never, rarely, sometimes, often, or always?”, as can be seen in Figure 1), and environmental risk factors, such as floor coverings or door sills, as stated in the German National Expert Standard Fall Prevention.

The results of the gait analysis and the questionnaire were computed into a score of 0-100 points, with a higher scoring indicating a higher fall risk.

The technical validity of LIN has been described elsewhere in several publications [12]. Thus, here, we provide a short summary.

The scientific approach underlying the app is based on a modular algorithm consisting of a video tester, a skeleton estimator (skeleton estimator 2D, skeleton estimator 3D, skeleton optimization 3D), and an analysis of mobility parameters. The skeleton estimator plays a central role. Both the validity of the mobility parameters and the validity of the analysis substantially depend on the spatial and temporal precision of the skeleton estimator [12].

### Reference Standards

Clinical guidelines recommend the evaluation of gait or balance disturbances to detect fall risk, but there is no gold standard for assessing the risk of falling in older adults measuring functional abilities [16]. However, there are several functional assessments available that have demonstrated good validity for identifying older people with a risk of falling. Three of the most widely used mobility assessments performed in therapeutic and nursing contexts are the Berg Balance Scale (BBS) [17], the Tinetti Test (TIN) [18], and the Timed Up & Go Test (TUG) [19]. In this study, these assessments were used as reference standards to evaluate functional mobility and balance.

TUG is a short-duration simple test on mobility [19], with a wide variety in clinical use. At the beginning of TUG, the participant sits on a chair, with arms placed on the armrests. On a command, the participant stands up, walks 3 m to a mark on the floor, turns around, and walks back to sit on the chair. TUG measures the time needed to complete the task in seconds. TUG is recommended as a routine screening test for falls in guidelines published by the American Geriatric Society and the British Geriatric Society [20] and has moderate-to-good sensitivity for predicting falls in older adults [16].

The BBS and TIN are scored based on a person’s ability to perform specific tasks. The BBS was developed in 1989 to determine balance stability among older adults [17]. Today, it is commonly used to measure balance in people with various disabilities and health conditions. The BBS consists of 14 items assessing static and dynamic components of mobility and balance ability on multiple levels, including standing, transitional movement, and a narrowed base of support. Each item is scored on a 5-point Likert scale from 0 to 4, with 0 indicating the lowest level of function and 4 the highest. The maximum score is 56, with higher scores indicating higher levels of functional mobility and lower risk of falling [21].

A score below 45 points indicates a higher risk of falls [22]. Based on a systematic review [23], the BBS has high interrater reliability with a pooled estimate of 0.97 (95% CI 0.96-0.98) and high intrarater reliability with a pooled estimate of 0.98 (95% CI 0.97-to 0.99). The BBS can differentiate between fallers and nonfallers in community-dwelling older people [23].

TIN, also called Performance-Oriented Mobility Assessment (POMA), is a clinical balance assessment tool originally developed for use with institutionalized patients. It measures both balance and gait performance. Several versions of TIN are available, with varying numbers of items and score ranges [24]. In the version used in our study, mobility is assessed with 8 items each for balance and gait performance. The items are scored on a 2-4-point Likert scale, with a maximum score of 28 points. A score below 19 points indicates a high risk of falls [18]. TIN showed good-to-excellent interrater and intrarater reliability (intraclass correlation coefficient [ICC]>0.80) in a cohort of 30 participants with Parkinson disease [25].

### Statistical Analysis

Baseline and sociodemographic data were collected, and Spearman rank correlation analysis was conducted.

Additionally limits of agreement (LOA) between LIN and TIN, the BBS, and TUG were evaluated using Bland-Altman (B-A) plots [26]. For plots with TIN and the BBS, we reversed the scale of LIN to adjust the direction of the scale to those of the reference scales. Next, we transformed results from TIN and the BBS into a ratio scale (0-100). As TUG and LIN both had the same direction of results (a higher score indicating a higher fall risk) and a transformation of TUG was not feasible, we performed all further steps with the original results obtained. Differences between each of the assessments and the results of LIN, as well as the mean of both respective measurements, and normal distributions of the differences of both observations were calculated. For this, we used the Shapiro-Wilk tests due to the relatively low number of participants, as well as visual inspection.

As the Shapiro-Wilk tests revealed mostly nonnormal distributions for the calculated differences between the measurements, we used the median and defined the upper and lower 95% of the sorted results as the threshold instead of the ±1.96 SD used for B-A plots with normal-distributed data. This approach was recommended by Bland and Altman [26] in their original publication on drawing B-A plots for nonnormal-distributed data sets.

Baseline and sociodemographic data as well as all correlation analyses were calculated using SPSS Statistics version 28 (IBM Corporation, Armonk, NY, USA). All B-A plots were drawn using Microsoft Excel 2016.

## Results

### Participant Characteristics

Data of 42 participants, with a mean age of 77.6 (SD 7.3) years were analyzed. As can be seen in Table 1, there was a higher percentage of female participants (n=25, 59.5%). In addition, 25 (59.5%) of the participants did not have a care level, and 26 (65%) of 40 participants did not have a level of disability based on the grading within the German health care system.

One participant was not able to perform TUG due to difficulty in rising from the chair. Additionally, in 3 cases, data from LIN could not be interpreted and had to be discarded. Therefore, all correlation analyses were performed and B-A plots drawn with 39 and 38 data sets, respectively.

As can be seen in Table 2, low scores for TUG indicated a high degree of functional mobility, while for the BBS and TIN, high scores indicated a high degree of mobility, and low scores for LIN indicated a low level of fall risk.

In Table 3, the correlations of the analogous fall risk and mobility assessments and LIN are presented.

Low scores for TUG indicated a high degree of functional mobility, while for the BBS and TIN, high scores indicated a high degree of mobility, and low scores for LIN indicated a low level of fall risk.

The results of LIN demonstrated a high correlation with the BBS (*r*_s_=–0.611) and a moderate-to-high correlation with TUG (*r*_s_=0.583) and TIN (*r*_s_=–0.563).

**Figure 2:**
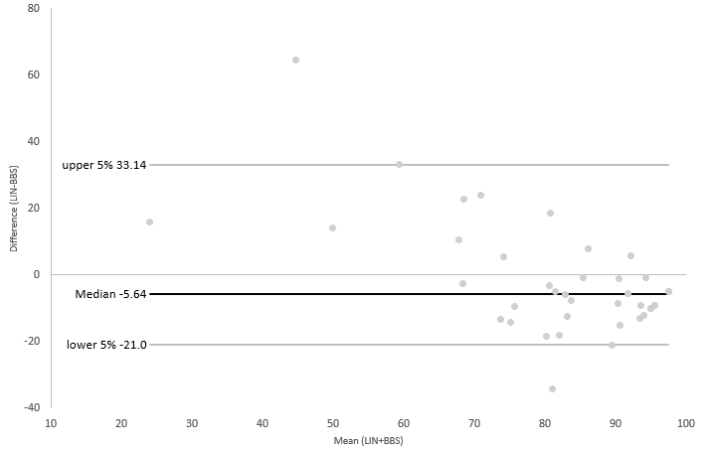
B-A plot of LIN and the BBS. B-A: Bland-Altman; BBS: Berg Balance Scale; LIN: Lindera Mobility Analysis.

**Figure 3:**
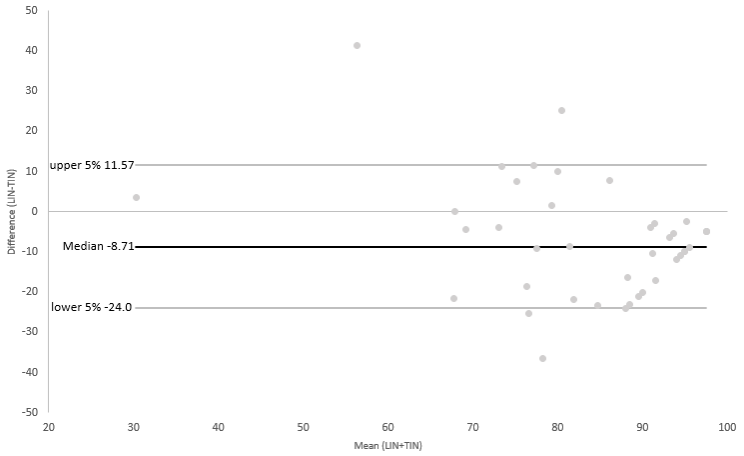
B-A plot of TIN and LIN. B-A: Bland-Altman; LIN: Lindera Mobility Analysis; TIN: Tinetti Test.

**Figure 4:**
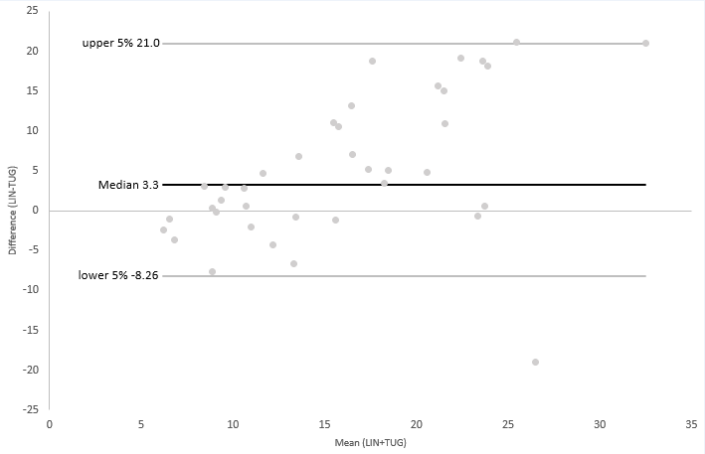
B-A plot of TUG and LIN. B-A: Bland-Altman; LIN: Lindera Mobility Analysis; TUG: Timed Up & Go Test.

As can be seen in Figures 2-4, the results of the nonparametric B-A plots revealed a median of differences of –8.71 (TIN), 5.64 (BBS), and 3.3 (TUG). Most data pairs were within the predefined LOA. Only 2 data pairs (5.1%) outside the LOA could be found for the BBS, while for TIN, 3 outliers could be observed (7.7%) and only 1 for TUG (2.6%). However, a proportional bias could be observed in all 3 plots based on a significant linear regression coefficient (0.014 for TIN and <0.001 for the BBS and TUG). Visual inspection of the 3 plots revealed a tendency for higher differences between measurements for all comparisons. Additionally, as can be seen in Figure 4, the B-A plot comparing LIN and TUG showed a visible trend of a negative difference between the 2 measurements for lower means, while demonstrating positive differences for higher means.

**Table 1:** Baseline data.

Characteristics		Participants
Age (years; N=42), mean (SD)		77.6 (7.3)
Female gender (N=42), n (%)		25 (59.5)
**Level of disability (N=40)^a^, n (%)**		
	No level	26 (65)
	<30	1 (2.5)
	31-60	8 (20.0)
	61-80	5 (12.5)
	>80	0
**Care level (N=42), n (%)**		
	0	25 (59.5)
	1	2 (4.7)
	2	7 (16.7)
	3	7 (16.7)
	4	1 (2.4)
	5	0

^a^The official level of disability is characterized by level 20 (low disability) up to level 100 (maximum disability)—§2 German Social Code (SGB) IX.

**Table 2:** Mobility data.

Assessment	Mean (SD)	Minimum	Maximum
TUG^a^ (N=40)	13.7 (5.8)	6.9	36
TIN^b^ (N=42)	23.9 (5.3)	8	28
BBS^c^ (N=42)	44.7 (13.0)	7	56
LIN^d^ (N=39)	19.8 (12.4)	5	68

^a^TUG: Timed Up & Go Test.

^b^TIN: Tinetti Test.

^c^BBS: Berg Balance Scale.

^d^LIN: Lindera Mobility Analysis.

**Table 3:** Spearman rank correlation of analog and digital fall risk and mobility assessment.

Assessment	TUG^a^			TIN^b^				LIN^c^		
	*r*_s_	*P* value	N	*r*_s_	*P* value	N	*r*_s_		*P* value	N
BBS^d^	–0.770^e^	.001	40	.730^e^	.001	42	–0.611^e^		.001	39
TUG	N/A^f^	N/A	N/A	–0.526^e^	.001	40	.583^e^		.001	38
TIN	N/A	N/A	N/A	N/A	N/A	N/A	–0.563^e^		.001	39

^a^TUG: Timed Up & Go Test.

^b^TIN: Tinetti Test.

^c^LIN: Lindera Mobility Analysis.

^d^BBS: Berg Balance Scale.

^e^The correlation was significant at the level of .01.

^f^N/A: not applicable.

## Discussion

### Principal Findings

The aim of this study was to evaluate the accuracy of LIN compared to reference standards for analog objective measures of older people’s fall risk. As our study shows, a moderate-to-high correlation according to Cohen [27] was found for LIN and the BBS, TIN and TUG. In this, the lowest correlation for the 3 reference assessments could be observed for LIN and TUG.

The results of our correlation analyses were verified by the B-A plots drawn. The B-A plots showed only a minority of the data pairs outside the predefined 95% limits. However, we observed a low-to-moderate proportional bias of the differences between results of LIN and the respective reference standards, indicating that both respective measurements might not be depicting the same construct. Moreover, we observed a skew in all plots, validating the observation of the correlation analyses. Due to the range and direction of the scales indicating a higher fall risk, we needed to transform our data for 2 plots in order to be able to obtain interpretable results. Additionally, as the differences between measurements were not normally distributed, we had to draw our B-A plots based on a nonparametric version.

This might have contributed to the results of the drawn plots. However, results from both correlation analyses and B-A plots could be interpreted as a sign that LIN can actually be superior in detecting older people at risk of falling compared to the 3 reference standards.

All 3 assessments are established tools for predicting falls in older people; however, none of them can be labeled as a gold standard. Although there might be different reasons for this, all of them have known flaws that have to be considered when planning to use any of them. As mentioned before, there are several versions available for TIN, making comparison between studies difficult. Additionally, both TIN and the BBS demonstrate only good but not high sensitivity and specificity for fall prediction in older adults living in care residence facilities [28]. The authors recommended using a combination of the BBS and a gait speed test in order to obtain more dependable results in this population. For TUG, Haines et al [29] found comparable problems in a population of older adults in a geriatric ward.

This merits some consideration. In contrast to TUG, LIN, TIN, and the BBS record complex movement sequences and thus evaluate balance, postural control, and gait symmetry.

In contrast, TUG merges all these functional requirements into 1 single information piece, the time needed to complete TUG. As a consequence, a lot of technology-based research aims at increasing the information value gathered through the relative easy-to-administer TUG, where TUG performance is often used to gather not only the TUG time but also the TUG stride length, as well as the forward und lateral tilt of the trunk and gait symmetry. Although TUG’s ability to predict falls in older adults has been established [19], several attempts have been made to increase the level of obtainable information while performing TUG, using video data and different sensor arrays [30-32]. All these studies have been, at least partly, successful in gathering information about gait and balance while performing TUG, but it still makes direct comparison between the original TUG and the expanded, technology-based versions difficult. In our study, LIN, in addition to information from a questionnaire and the time to complete TUG, measured other factors, such as stride length and the forward and lateral tilt of the trunk and gait symmetry. As stated before, TIN and the BBS evaluate complex movement sequences that resemble a wide variety of everyday activities and thus test a participant’s balance, postural control, and gait symmetry. Therefore, the gathered data seem to be more comparable to more complex (and time-demanding) assessments, such as the BBS and TIN. This is, in our opinion, reflected in the high correlation coefficients between LIN and the BBS and TIN in contrast to the more modest correlation with TUG.

Additionally, LIN uses an additional questionnaire based on the German National Expert Standard Fall Prevention and as such provides a guideline for the prevention of falls [14]. The questionnaire encompasses items about not only intrinsic factors, such as comorbidities, incontinence, fear of falling, and prior falls, but also extrinsic factors, such as mobility aids, barriers in the living environment, shoes used at home, and several other factors that have been identified as contributors to the risk of falling. Therefore, LIN includes, in comparison to the functional assessments of gait and balance that are recommended in geriatrics and were used in this study, more dimensions of the phenomenon of falls in older adults and is, thus, in our opinion, more comprehensive that a purely functional assessment for identifying patients with fall risk. Whether this leads to any potential superiority of LIN cannot be answered based on the available data. For this, additional research is necessary that includes the prospective establishment of diagnostic criteria as well as its ability to prevent falls. We conclude therefore that for gaining deeper insight into the potential of technology-based mobility and fall risk assessments, more detailed comparators are needed.

Despite these limitations, we deem our results satisfactory. The low number of data pairs outside the LOA indicate, in our estimation, a satisfactory level of comparability of the results of LIN with our reference standards. The observable bias in all 3 plots is, in our estimation, acceptable. Due to the reason stated before insofar, a complete agreement between the measurements cannot be expected. However, we are aware of the fact that the results presented here have to be interpreted with caution and have to be verified in further studies.

Compared to other apps for fall risk analysis, such as FallSA [33], LIN showed a slightly higher significant correlation with the established BBS. FallSA was significant moderately correlated (*r*=0.518, *P*<.001) with the Physical Profile Assessment [11]. In 2021, iPhone manufacturer Apple Inc. offered a function in the current version of its iOS (iOS 15) that is supposed to prevent falls. As the manufacturer stated, “Walking Steadiness on iPhone is a first-of-its-kind health metric that can give you insight into your risk of falling. It uses custom algorithms that assess your balance, strength, and gait” [34]. Based on calculated gait stability, the software is supposed to predict the risk of falling. Both FallSA and iOS 15 measure functional ability. In contrast, LIN is based on the measurement of functional ability and surveying intrinsic factors of its users. Furthermore, the FallSA app as well as iOS 15 are not specifically labeled as medical devices in the sense of the European Medical Device Regulation—Regulation (EU) 2017/745 of the European Parliament and of the Council of 5 April 2017 on Medical Devices. In contrast, LIN is a class 1 medical device. Being a medical device allows professionals involved in care, such as nurses, physical therapists, and physicians, to use the results of the app to assist their nursing appraisals or diagnoses.

Using LIN or other medical devices with the ability to identify fall risks in older people while involving health professionals offers great potential. In 2021, Meekes et al [35] studied the level of information general practitioners (GPs) had available for any of their patients with frailty about their fall history as well as the occurrence of fear of falling. In their study, GPs had no information about fall history in 668 (48%) of the affected patients [35].

Additionally, as several studies have demonstrated that a significant portion of patients tend to underestimate their own fall risk [36,37], the LIN app offers high potential for determining one’s own fall risk as a nonprofessional. This gives older people an opportunity to self-assess their own fall risk and, with repeated measurements, any changes in their fall risk status over time.

### Conclusion

Using LIN has the potential to enable older people to be more independent of the initial determination of a fall risk by GPs or other health care professionals and also enables them to identify and respond to positive or negative changes in their own fall risk. This provides older adults with the ability to manage their own fall risk in an effective and adequate manner. Using LIN can help reduce fall events in people aged 65 years or more. Further study is indicated to verify validity.

## Abbreviations

B-A: Bland-Altman
BBS: Berg Balance Scale
GP: general practitioner
LIN: Lindera Mobility Analysis
LOA: limits of agreement
SGB: German Social Code
TIN: Tinetti Test
TUG: Timed Up & Go Test

## References

[1] Todd C, et al World Health Organisation Global Report on Falls Prevention in Older Age/Citation Formats.

[2] Haagsma JA, Olij BF, Majdan M, van Beeck EF, Vos T, Castle CD, Dingels ZV, Fox JT, Hamilton EB, Liu Z, Roberts NLS, Sylte DO, Aremu O, Bärnighausen TW, Borzì AM, Briggs AM, Carrero JJ, Cooper C, El-Khatib Z, Ellingsen CL, Fereshtehnejad S, Filip I, Fischer F, Haro JM, Jonas JB, Kiadaliri AA, Koyanagi A, Lunevicius R, Meretoja TJ, Mohammed S, Pathak A, Radfar A, Rawaf S, Rawaf DL, Riera LS, Shiue I, Vasankari TJ, James SL, Polinder S (2020). Falls in older aged adults in 22 European countries: incidence, mortality and burden of disease from 1990 to 2017. Inj Prev.

[3] Choi NG, Choi BY, DiNitto DM, Marti CN, Kunik ME (2019). Fall-related emergency department visits and hospitalizations among community-dwelling older adults: examination of health problems and injury characteristics. BMC Geriatr.

[4] Deshpande N, Metter EJ, Lauretani F, Bandinelli S, Guralnik J, Ferrucci L (2008). Activity restriction induced by fear of falling and objective and subjective measures of physical function: a prospective cohort study. J Am Geriatr Soc.

[5] Hoffman GJ, Rodriguez HP (2015). Examining contextual influences on fall-related injuries among older adults for population health management. Popul Health Manag.

[6] Lippert T, Maas R, Fromm MF, Luttenberger K, Kolominsky-Rabas P, Pendergrass A, Gräßel E (2020). [Impact of sedating drugs on falls resulting injuries among people with dementia in a nursing home setting]. Gesundheitswesen.

[7] Yeung SS, Reijnierse EM, Pham VK, Trappenburg MC, Lim WK, Meskers CG, Maier AB (2019). Sarcopenia and its association with falls and fractures in older adults: a systematic review and meta-analysis. J Cachexia Sarcopenia Muscle.

[8] Unhjem R, van den Hoven LT, Nygård M, Hoff J, Wang E (2019). Functional performance with age: the role of long-term strength training. J Geriatr Phys Ther.

[9] Pérez-Ros P, Martínez-Arnau FM, Orti-Lucas RM, Tarazona-Santabalbina FJ (2019). A predictive model of isolated and recurrent falls in functionally independent community-dwelling older adults. Braz J Phys Ther.

[10] Kakulla BN 2020 Tech Trends of the 50+.

[11] Singh DKA, Goh JW, Shaharudin MI, Shahar S (2021). A mobile app (FallSA) to identify fall risk among Malaysian community-dwelling older persons: development and validation study. JMIR Mhealth Uhealth.

[12] Azhand A, Rabe S, Müller S, Sattler I, Heimann-Steinert A (2021). Algorithm based on one monocular video delivers highly valid and reliable gait parameters. Sci Rep.

[13] Stamm O, Heimann-Steinert A (2020). Accuracy of monocular two-dimensional pose estimation compared with a reference standard for kinematic multiview analysis: validation study. JMIR Mhealth Uhealth.

[14] Schmidt S (2016). Expertenstandard Sturzprophylaxe in der Pflege. Expertenstandards in der Pflege - eine Gebrauchsanleitung.

[15] Büscher A, Möller A (2014). Deutsches Netzwerk für Qualitätsentwicklung in der Pflege (DNQP): Aktueller Stand. Public Health Forum.

[16] Nordin E, Lindelöf N, Rosendahl E, Jensen J, Lundin-Olsson L (2008). Prognostic validity of the Timed Up-and-Go test, a modified Get-Up-and-Go test, staff's global judgement and fall history in evaluating fall risk in residential care facilities. Age Ageing.

[17] Berg K (1989). Measuring balance in the elderly: preliminary development of an instrument. Physiother Can.

[18] Tinetti ME (1986). Performance-oriented assessment of mobility problems in elderly patients. J Am Geriatr Soc.

[19] Podsiadlo D, Richardson S (1991). J Am Geriatr Soc.

[20] Panel on Prevention of Falls in Older Persons‚ American Geriatrics SocietyBritish Geriatrics Society (2011). Summary of the Updated American Geriatrics Society/British Geriatrics Society clinical practice guideline for prevention of falls in older persons. J Am Geriatr Soc.

[21] Lajoie Y (2013). Effect of computerized feedback postural training on posture and attentional demands in older adults. Aging Clin Exp Res.

[22] Berg KO, Wood-Dauphinee SL, Williams JI, Maki B (1992). Measuring balance in the elderly: validation of an instrument. Can J Public Health.

[23] Marques A, Almeida S, Carvalho J, Cruz J, Oliveira A, Jácome C (2016). Reliability, validity, and ability to identify fall status of the balance evaluation systems test, mini-balance evaluation systems test, and brief-balance evaluation systems test in older people living in the community. Arch Phys Med Rehabil.

[24] Köpke S, Meyer G (2006). The Tinetti test: Babylon in geriatric assessment. Z Gerontol Geriatr.

[25] Kegelmeyer DA, Kloos AD, Thomas KM, Kostyk SK (2007). Reliability and validity of the Tinetti Mobility Test for individuals with Parkinson disease. Phys Ther.

[26] Bland JM, Altman DG (1999). Measuring agreement in method comparison studies. Stat Methods Med Res.

[27] Cohen J (1982). Set correlation as a general multivariate data-analytic method. Multivariate Behav Res.

[28] Harada N, Chiu V, Damron-Rodriguez J, Fowler E, Siu A, Reuben DB (1995). Screening for balance and mobility impairment in elderly individuals living in residential care facilities. Phys Ther.

[29] Haines T, Kuys SS, Morrison G, Clarke J, Bew P (2008). Balance impairment not predictive of falls in geriatric rehabilitation wards. J Gerontol A Biol Sci Med Sci.

[30] Botolfsen P, Helbostad JL, Moe-Nilssen R, Wall JC (2008). Reliability and concurrent validity of the Expanded Timed Up-and-Go test in older people with impaired mobility. Physiother Res Int.

[31] Dibble LE, Lange M (2006). Predicting falls in individuals with Parkinson disease: a reconsideration of clinical balance measures. J Neurol Phys Ther.

[32] Fudickar S, Kiselev J, Frenken T, Wegel S, Dimitrowska S, Steinhagen-Thiessen E, Hein A (2020). Validation of the ambient TUG chair with light barriers and force sensors in a clinical trial. Assist Technol.

[33] Lord SR, Menz HB, Tiedemann A (2003). A physiological profile approach to falls risk assessment and prevention. Phys Ther.

[34] Apple (Deutschland) iOS - Gesundheit.

[35] Meekes WMA, Leemrijse CJ, Weesie YM, van de Goor IAM, Donker GA, Korevaar JC (2021). Falls prevention at GP practices: a description of daily practice. BMC Fam Pract.

[36] Braun BL (1998). Knowledge and perception of fall-related risk factors and fall-reduction techniques among community-dwelling elderly individuals. Phys Ther.

[37] Moreira MN, Bilton TL, Dias RC, Ferriolli E, Perracini MR (2017). What are the main physical functioning factors associated with falls among older people with different perceived fall risk?. Physiother Res Int.

